# Attachment Style and Jealousy in the Digital Age: Do Attitudes About Online Communication Matter?

**DOI:** 10.3389/fpsyg.2021.678542

**Published:** 2021-07-16

**Authors:** Kieran T. Sullivan

**Affiliations:** Department of Psychology, Santa Clara University, Santa Clara, CA, United States

**Keywords:** jealousy, dating relationships, online communication, social media, attachment, attitudes

## Abstract

Romantic jealousy, a complex response to a real or perceived threat to a romantic relationship, can have serious negative consequences for individuals, partners and perceived rivals. The likelihood of a jealous response is heightened among individuals who experience attachment anxiety, and online communication and social media provide unique fodder for romantic jealousy. The purpose of the current study is to test whether the association between attachment anxiety and online jealousy (jealous response to ambiguous hypothetical online scenarios) is moderated by negative attitudes about online communication. Individuals in dating relationships were asked about attachment anxiety and attitudes about online communication (i.e., apprehension and concern about misunderstandings) as well as emotional, cognitive, and behavioral online jealousy. Hierarchical linear regression revealed an attachment anxiety-attitude interaction, such that the link between attachment anxiety and jealousy was stronger for participants with relatively low levels of negative attitudes about online communication compared to participants with relatively high levels of negative attitudes. The current study expands knowledge about attachment anxiety and jealousy in the context of online communication and social media, and highlights the importance of considering attitudes about online communication when studying relationship processes in the digital arena.

## Introduction

The association between attachment style and romantic jealousy is well-established (Dandurand and Lafontaine, 2014; see Martinex-Leon et al., 2017 for a recent review) and jealousy, a complex response to a real or perceived threat to an intimate relationship, can have negative, serious – even fatal – consequences for individuals, partners and perceived rivals (Mužinié et al., 2003). In the digital age, the ease with which individuals can access information about and monitor their partners (Rus and Tiemensma, 2017), along with the ubiquity of social media, makes the importance of understanding processes related to jealousy in the digital arena clear.

### Attachment Anxiety and Jealousy

Some individuals are more likely to respond to real or perceived threats with jealousy – across partners and situations – than others. Considerable evidence points to attachment anxiety – anxiety about being abandoned or rejected by one’s relationship partner – as a key predictor of offline jealous response (Dandurand and Lafontaine, 2014; Martinex-Leon et al., 2017). Attachment theory posits that attachment styles are formed based on early experiences with caregivers (Bowlby, 1988). Caregiver responsiveness leads individuals to develop working models of the self (I am worthy of love/I am not worthy of love) and others (Others are trustworthy/Others are not trustworthy). When early caregivers respond erratically to a child’s needs, she develops an insecure attachment style, believing that she is not worthy of love and/or that others are not dependable (Ainsworth et al., 1978). These working models are quite stable, and ample evidence suggests they affect intimate relationships in adulthood (Collins and Read, 1990; Simpson, 1990). Adult attachment style is commonly characterized using two dimensions, anxiety and avoidance (Bartholomew and Horowitz, 1991); attachment anxiety refers to high scores on the anxiety dimension, which may be further categorized as preoccupied/anxious (when avoidance is low) and fearful (when avoidance is high).

Research on adult attachment has shown that those high in attachment anxiety are more likely to monitor partner behavior as they seek assurance of continued interest, and are more likely to perceive emotional and sexual threats to their relationships (White and Mullen, 1989). They experience jealousy more frequently and intensively than avoidant and securely attached individuals, and respond to jealousy-provoking situations with more fear, anger, and sadness (Sharpsteen and Kirkpatrick, 1997; Guerrero, 1998; Rodriguez et al., 2015; Tani and Ponti, 2016).

Attachment style is differentially associated with specific jealousy components, often conceptualized as emotional (affective responses to perceived threats), cognitive (thoughts, suspicions, and worries about a partner’s extradyadic behaviors), and behavioral (behavioral reactions to jealousy-evoking situations, such as checking, snooping, and surveillance; Pfeiffer and Wong, 1989). Some studies have found associations between attachment anxiety and cognitive and behavioral jealousy, but not emotional jealousy (e.g., Rydell and Bringle, 2007). Other studies have found attachment anxiety to be related to all three components of jealousy (e.g., Elphinston et al., 2011; Rodriguez et al., 2015), though the strength of associations varied, with the association between attachment anxiety and cognitive and behavioral jealousy being about twice as strong as the association between attachment anxiety and emotional jealousy (Rodriguez et al., 2015; Bevan, 2017).

### Attachment Anxiety and Online Jealousy

The affordances of social media allow people high in attachment anxiety new ways to manifest their fear of abandonment (e.g., making relationship status public and highly visible) and to reassure themselves of their partners’ continuing love and fidelity (e.g., monitoring partners’ activities). These affordances provide unparalleled access to information about romantic partners [via posts, pictures, location tracing, and granting or restricting access to information (e.g., enabling read receipts); Muscanell et al., 2013; Muise et al., 2014]; further, the information gleaned from social media can be ambiguous and open to interpretation, making it easier for individuals high in attachment anxiety to interpret information as threatening to their relationships.

Although distinct from offline jealousy, findings from studies of online jealousy and attachment anxiety echo findings from studies of offline jealousy (Muise et al., 2014). In two studies of attachment style and Facebook-related jealousy and surveillance, attachment anxiety was associated with higher Facebook jealousy and more surveillance cross-sectionally, and more surveillance over a one-week period (Marshall et al., 2013; Hira and Bhogal, 2020). In the context of viewing pictures of romantic partners touching an opposite-sex friend, individuals high in preoccupied/anxious attachment reported higher levels of fear and anger compared to individuals low in attachment anxiety (Miller et al., 2014). In response to ambiguous, potentially threatening Facebook content on a partner’s wall, individuals higher in preoccupied/anxious attachment were more likely to experience negative emotions, such as fear, worry, and jealousy (Fleuriet et al., 2014). Individuals with attachment anxiety were more likely to use Facebook to increase relationship visibility (e.g., reporting relationship statues; Emery et al., 2014); and individuals high in preoccupied/anxious and fearful attachment expressed more uncertainty about their relationships, and engaged in more interpersonal electronic surveillance (Fox and Warber, 2014) and jealousy induction (Wegner et al., 2018).

These findings shed light on the association between attachment anxiety and jealousy in the digital arena, however, unique variability in the context of online communication and social media remains to be fully explored. In their review of research on social network site use and romantic relationships, Rus and Tiemensma (2017) concluded that the impact of individual difference variables, such as attachment style, on romantic jealousy may be amplified or mitigated in the online environment, and subsequent research suggests that the strength of the association between attachment anxiety and online jealousy may vary based on individual and relationship factors (e.g., Wegner et al., 2018).

A key variable in understanding how and to what extent individuals react to ambiguous online information with jealousy may be individuals’ attitudes about the medium (e.g., concern about misunderstandings). Online communication attitudes are conceptualized as cognitive and affective orientations toward online communication (Ledbetter et al., 2011). Negative orientations include apprehension about online communication and concern that online communication will lead to misunderstandings (Ledbetter, 2009; Bernhold and Rice, 2020). While there is scant research examining online communication attitudes and romantic jealousy, there is evidence that the way individuals think and feel about communication via digital media strengthen or inhibit the impact of communication variables (e.g., communication goals, frequency of communication) on relationship variables (e.g., relational closeness; e.g., Ledbetter and Mazer, 2014; Bernhold and Rice, 2020). In this paper, we propose that the strength of the association between attachment anxiety and online jealousy will depend, in part, on individuals’ negative attitudes about online communication with their romantic partners.

### Purpose and Hypotheses

The purpose of the current study is to replicate and extend the findings regarding the association between attachment anxiety and online jealousy and to evaluate whether this association varies based on attitudes about online communication. Based on previous research, we predict that attachment anxiety will be positively associated with online jealousy (e.g., Muise et al., 2014), and that these associations will be stronger for cognitive and behavioral jealousy compared to emotional jealousy (e.g., Rydell and Bringle, 2007). Given the lack of previous research on online attitudes and jealousy, we make no specific predictions about associations between negative online attitudes (i.e., concern about misunderstandings and apprehension about using online communication) and online jealousy, nor do we have specific predictions as to the nature of any interaction between attachment anxiety and negative online attitudes. A significant interaction, if found, may take the form of a potentiating model wherein negative attitudes strengthen the association between attachment anxiety and online jealousy; that is, the correlation between attachment anxiety and jealousy will be stronger for participants who report relatively high concern about potential misunderstandings and apprehension about online communication, compared to those report relatively little apprehension or concern. Alternatively, an interaction may take the form of a mitigating model, wherein strong negative attitudes weaken the association between attachment anxiety and jealousy; that is, the correlation between attachment anxiety and jealousy will be weaker for participants who report relatively high concern about potential misunderstandings and apprehension about online communication, compared to those report relatively little apprehension and concern.

There is some evidence of gender differences in online jealousy and in the association between attachment anxiety and online jealousy (e.g., Muise et al., 2009; Emery et al., 2014; Wegner et al., 2018) so gender differences in online jealousy and attitudes are also examined, as well as gender differences in the association between attachment anxiety and online jealousy.

## Materials and Methods

### Participants

Participants were drawn from a university on the west coast of the United States. General Psychology students who were in dating relationships (*N* = 84^1^) were recruited via a participation pool. Participants were White (51.2%), Asian-American (26.2%), Latinx (22.6%), African-American (3.6%), and Native American (0.01%); 69% were women. The mean relationship length was 14.9 months (SD = 13.1). Two participants were engaged; none were married. Only one participant reported that she was currently living with her dating partner. Three participants reported being in same-sex relationships. Participants received course credit for participation, which was not mandatory; an alternative assignment was available for students to earn equivalent credit for their general psychology course.

### Procedure

Before beginning the study, institutional review board approval was obtained. All students in general psychology classes were invited to log onto a participation pool website. Students who indicated they were in a dating relationship were invited to sign up for a lab session; there were no additional eligibility requirements. Participants read an informed consent form that explained the study and indicated that they could withdraw from the study at any time and/or skip any questions and still receive course credit for participating. These points were reiterated verbally by researchers. No participants withdrew from the study. Participants filled out a series of online questionnaires assessing demographics, attachment style, attitudes toward online communication, as well as emotional, cognitive and behavioral jealousy related to hypothetical online scenarios.

### Measures

**Attachment anxiety.** Attachment anxiety was assessed using the anxiety subscale of the Revised Adult Attachment Scale (Collins and Read, 1990). Participants were instructed to “rate the extent to which each item describes you and your feelings about romantic relationships. Think about all your romantic relationships (past and present) and respond in terms of how you generally feel in these relationships.” Participants responded to each item on a scale of 1 (does not describe me at all) to 5 (describes me very well). An example item is “In relationships, I often worry that my partner does not really love me.” Coefficient alpha for this scale was 0.77.

**Online Jealousy**. Online jealousy was assessed by adapting questions from the Facebook Jealousy Scale (FJS; Muise et al., 2009) to refer to social networking sites generally as well as personal messaging (see Sullivan and Bruchmann, unpublished, for a psychometric analysis of a similar measure adapted from the FJS). For items assessing emotional jealousy participants were instructed to rate how they would feel in various hypothetical online situations, from 1 (not upset) to 7 (very upset) for the emotional subscale (10 items; e.g., “Your partner posted/sent a message to someone of the preferred sex”). For items assessing cognitive and behavioral jealousy participants were instructed to rate “how likely they were to do each of the following.” Four items assessed cognitive jealousy (e.g., “Worry that your partner is using social media to reconnect with past romantic or sexual partners?”) and six items assessed behavioral jealousy (e.g., “Monitor your partner’s social media activity?”) Participants responded to each item a scale from 1 (very unlikely) to 7 (very likely). Coefficient alpha for the emotional, cognitive, and behavioral jealousy scales were 0.91,0.79, and 0.81, respectively.

**Attitudes about communicating online with dating partner.** Attitudes about communicating online were assessed by adapting the misunderstanding (5 items) and apprehension (8 items) subscales of the Online Attitudes Questionnaire (OAQ) developed by Ledbetter (2009). The OAQ was developed to assess interpersonal relationships generally; we adapted the questions to refer specifically to dating partners. Participants responded to each item on a scale of 1 (strongly disagree) to 7 (strongly agree). Example items include “It is easy to take meanings that my partner did not intend when reading online messages” (misunderstandings), “I feel tense and nervous when communicating with my partner online” (apprehension). Coefficient alphas for the subscales were 0.89 (misunderstanding) and 0.86 (apprehension).

## Results

Descriptive statistics and correlations among all variables can be seen in Table 1. As expected, the correlations between attachment anxiety and jealousy subscales were positive and significant. Fisher *r* to *z* transformations were used to determine whether the associations between attachment anxiety and jealousy were significantly higher cognitive (*r* = 0.37) and/or behavioral (*r* = 0.36) jealousy than the association between attachment anxiety and emotional jealousy (*r* = 0.26). No significant differences were found, *z* = −0.75, *p* = 0.24 (emotional vs. cognitive jealousy); *z* = −0.68, *p* = 0.24 (emotional vs. behavioral jealousy). The attitude subscales, misunderstanding and apprehension, were positively related to the jealousy subscales and the jealousy variables were positively related to one another. Cognitive and behavioral jealousy were highly correlated with one another, calling into question how distinct these two components were; thus, the total score of items assessing cognitive and behavioral subscales was used for all subsequent analyses. The online attitude subscales were positively related to attachment anxiety and to one another.

**TABLE 1:** Descriptive statistics and correlations among all variables.

Variables	Range	*M*	*SD*	1	2	3	4	5
(1) Attachment Anxiety	7–28	15.36	4.41					
**Online Jealousy**								
(2) Jealous Emotions	10–64	31.85	12.10	0.26*				
(3) Jealous Thoughts	4–19	8.23	4.77	0.37**	0.5***			
(4) Jealous Behaviors	6–28	12.02	6.31	0.36**	0.5***	0.77***		
**Attitudes About Online Communication**								
(5) Concern About Misunderstanding	5–35	20.67	7.84	0.32**	0.32**	0.37**	0.47***	
(6) Apprehension	17–56	22.58	9.88	0.48***	0.4***	0.38**	0.38*	0.54***

*p < 0.05; **p < 0.01; ***p < 0.001.

Gender differences in attitudes and online jealousy were assessed using a one-way analysis of variance (ANOVA). No significant gender differences were found for concern about misunderstandings (*t* = −0.41, *p* = 0.59) or apprehension (*t* = 0.49, *p* = 0.16) nor for emotional (*t* = 0.88, *p* = 0.42) or cognitive/behavioral (*t* = −0.89, *p* = 0.38) jealousy. Fisher *r* to *z* transformations were used to compare correlation coefficients between attachment anxiety and jealousy for men and women; no significant differences were found for emotional jealousy (*z* = 0.04, *p* = 0.12) or for cognitive/behavioral jealousy (*z* = 0.54, *p* = 0.42).

Four hierarchical linear regression models were run to assess 1) whether attachment anxiety and the attitudes scales were significantly associated with emotional and/or cognitive/behavioral jealousy (main effects) and whether the attitudes scales moderated the relationship between attachment anxiety and the jealousy subscales (see Table 2). First, attachment anxiety and attitude (misunderstanding or apprehension) were entered as a block (Step 1), then the relevant interaction term was entered (Step 2). To reduce multicollinearity, all variables were centered for these analyses (Aiken and West, 1991).

**TABLE 2:** Hierarchical linear regression analyses of anxious attachment and online attitudes predicting jealousy.

		Adjusted						Adjusted					
Models		B	SE B	*β*	ΔF	r2	ΔR^2^	B	SE B	*β*	ΔF	r2	ΔR^2^
		
		Jealous Feelings						Jealous Cognitions and Behavior					
**Misunderstandings**													
Step 1					5.91**	0.11					15.3***	0.26	
	Attachment Anxiety	0.51	0.31	0.18				0.63	0.24	0.27***			
	Misunderstandings	0.39	0.17	0.25*				0.49	0.13	0.37***			
Step 2					5.03*	0.15	0.05*				9.32**	0.33	0.08**
	AnxiousxMisunderstandings	0.08	0.04	1.2*				0.08	0.03	1.5**			
**Apprehension**													
Step 1					7.71**	0.14					10.79***	0.19	
	Attachment Anxiety	0.25	0.33	0.09				0.6	0.27	0.25*			
	Apprehension	0.43	0.15	0.35**				0.3	0.12	0.28*			
Step 2					3.62#	0.17	0.04#				0	0.18	0
	AnxietyxApprehension	0.06	0.03	1.12#				0	0.03	0.02			

^#^p < 0.10; *p < 0.05; **p < 0.01; ***p < 0.001.

### Emotional Jealousy

**FIGURE 1:**
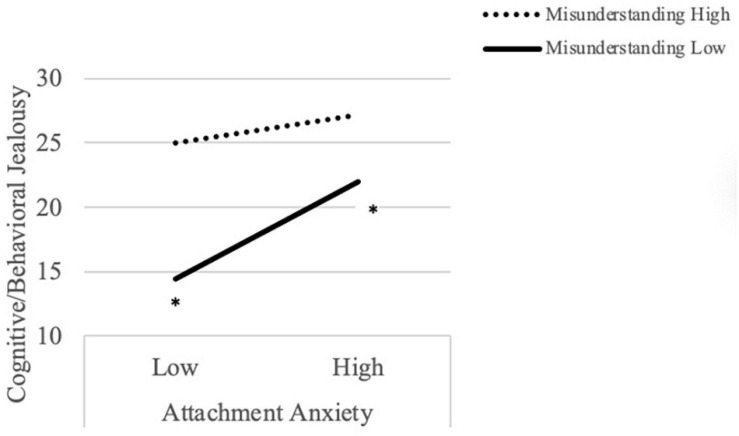
Attachment style and concern about misunderstandings interact to predict cognitive/behavioral jealousy.

Regarding emotional jealousy and concern about misunderstandings, there was no main effect of attachment anxiety on emotional jealousy, however, there was a significant main effect of concern about misunderstandings, such that individuals with relatively high levels of concern reported higher levels of jealousy compared to those with relatively low levels of concern, regardless of level of attachment anxiety. There was also a significant interaction between attachment anxiety and concern about misunderstandings. To explore the interaction, emotional jealousy scores were plotted for each of the variables at one standard deviation below and above the mean (see Figure 1). Simple slopes analyses were conducted to test the significance of differences in emotional jealousy (Baron and Kenny, 1986; Holmbeck, 2002); results indicated that, among individuals relatively low in concern, emotional jealousy was significantly higher for participants with relatively high levels of attachment anxiety compared to those with relatively low levels of attachment anxiety, *t* = 2.70, *p* < 0.01. When concern was relatively high, however, there was no significant difference between participants high in attachment anxiety compared to those low in attachment anxiety, *t* = −0.65, *p* = 0.31.

**FIGURE 2:**
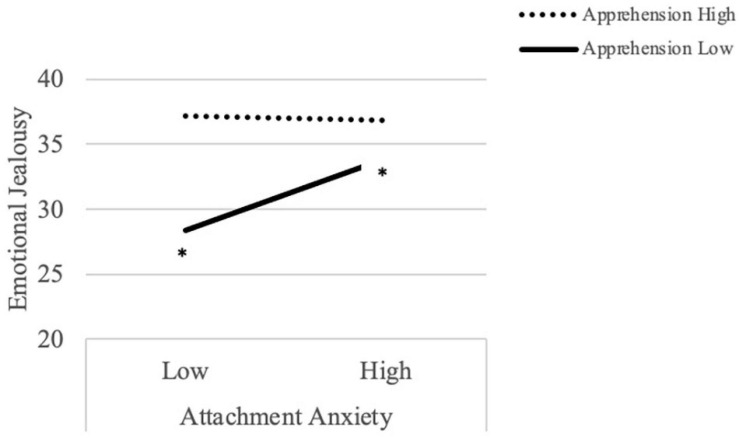
Attachment style and apprehension about communicating online interact to predict emotional jealousy.

Similarly, regarding emotional jealousy and apprehension about online communication, no main effect of attachment anxiety was found, but there was a significant main effect of apprehension wherein apprehension was positively related to emotional jealousy, regardless of level of attachment anxiety. There was a marginal interaction effect (*p* = 0.062) and simple slopes analysis confirmed the same pattern found in the misunderstandings model (Figure 2); that is, for individuals low in apprehension, emotional jealousy was significantly higher for participants with high levels of attachment anxiety compared to those with relatively low levels of attachment anxiety, *t* = 2.48, *p* < 0.05. When apprehension was high, however, there was no significant difference between participants high in attachment anxiety compared to those low in attachment anxiety, *t* = 0.11, *p* = 0.91.

### Cognitive/Behavioral Jealousy

**FIGURE 3:**
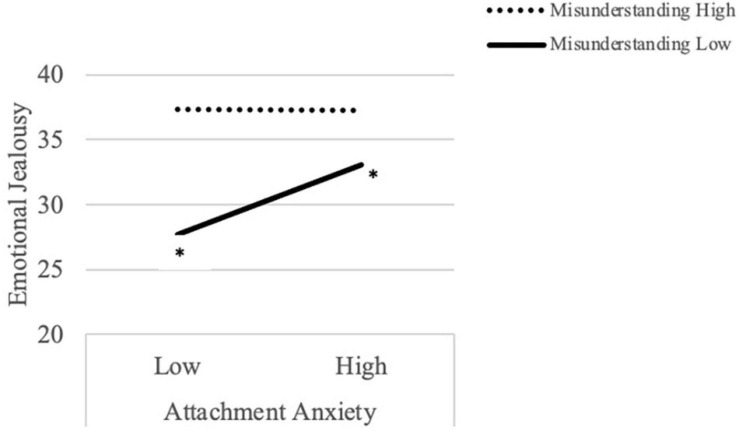
Attachment style and concern about misunderstandings interact to predict emotional jealousy.

Regarding cognitive/behavioral jealousy and concern about misunderstandings, there was a significant main effect of attachment anxiety on cognitive/behavioral jealousy such that individuals relatively high in attachment anxiety reported higher levels of cognitive/behavioral jealousy compared to individuals relatively low in attachment anxiety, regardless of level of attachment anxiety. There was also a significant main effect of concern about misunderstandings, such that individuals with relatively high levels of concern reported higher levels of cognitive/behavioral jealousy compared to those with relatively low levels of concern, regardless of level of attachment anxiety. There was also a significant interaction, similar to that found with emotional jealousy models (Figure 3); simple slopes analyses revealed that, among individuals with low levels of concern, emotional jealousy was significantly higher for participants with relatively high levels of attachment anxiety compared to those with relatively low levels of attachment anxiety, *t* = 4.52, *p* < 0.001. When concern was high, however, there was no significant difference between participants high in attachment anxiety compared to those low in attachment anxiety, *t* = −0.88, *p* = 0.45.

Finally, regarding cognitive/behavioral jealousy and apprehension, there were main effects of attachment anxiety and apprehension wherein attachment anxiety was positively related to cognitive/behavioral jealousy, regardless of level of apprehension, and apprehension was positively related to cognitive/behavioral jealousy, regardless of level of attachment anxiety. There was no significant interaction between attachment anxiety and apprehension.

## Discussion

### Summary and Implications

The current findings replicate and extend previous findings about attachment anxiety and online jealousy. Consistent with predictions and past studies (e.g., Marshall et al., 2013; Miller et al., 2014; Wegner et al., 2018), zero-order correlations indicated that attachment anxiety was related to online jealousy. Contrary to predictions, zero-order correlations between attachment anxiety and cognitive and behavioral online jealousy were not significantly higher than correlations between attachment anxiety and emotional online jealousy. This is contradictory to previous findings that suggest that attachment anxiety correlates more strongly with cognitive and behavioral jealousy than with emotional jealousy (e.g., Rydell and Bringle, 2007). Notably, however, the current study found no main effect of attachment anxiety on emotional jealousy in regression analyses, but did find a significant main effect of attachment anxiety on cognitive/behavioral jealousy. These main effects and the interaction effects (discussed presently) echo findings by Rodriguez et al. (2015) that trust did not moderate the association between attachment anxiety and emotional jealousy, but did moderate the associations between attachment anxiety and cognitive and behavioral jealousy. Further, distrust was more strongly related to cognitive jealousy when attachment anxiety was high, compared to when attachment anxiety was low, and distrust was only related to behavioral jealousy when attachment anxiety was high. Based on these findings, the researchers speculated that “it may be more natural to experience cognitions associated with jealousy when experiencing lower levels of trust in one’s partner, but it is less natural to act on those thoughts” (pp. 310). At the very least, evidence presented here as well as evidence from previous studies indicate that nuanced models will be necessary to fully account for variance in attachment anxiety and jealousy feelings, thoughts, and behaviors (see also Rydell and Bringle, 2007). Null results regarding gender differences in the current study are consistent with some, but not all, past research (e.g., Muise et al., 2009; Emery et al., 2014; Wegner et al., 2018) thus more nuanced models of gender differences may be required as well. Regarding exploratory analyses of attitudes about online communication, concern about misunderstandings and apprehension about communicating online were associated with higher levels of online emotional and cognitive/behavioral jealousy, and the strength of the association between attachment anxiety and online jealousy depended, in part, on online attitudes. These findings are most consistent with a mitigating model wherein the association between attachment anxiety and jealousy appears to be diminished by strong negative attitudes about online communication. That is, the impact of attachment anxiety on jealous responses is lower for individuals who are very concerned about misunderstandings when communicating online with dating partners, compared to those relatively unconcerned about misunderstandings in online communications. There is some evidence, although marginal, that this holds true for apprehension about online communication in the context of emotional jealousy, though not for cognitive/behavioral jealousy.

At this point, however, given the correlational nature of the design, construing that negative attitudes affect the impact of attachment anxiety on jealousy is speculative. It may be, for example, that jealousy is driving attitudes about online communication. Further, given the lack of previous research examining the associations among attachment anxiety, negative online attitudes and jealousy, replication of these findings is critical before accepting these findings – and their implications – with confidence. Keeping these important limitations in mind, we offer some initial thoughts about what the current finding may imply. If we begin with the assumption that attachment anxiety does indeed affect jealous responses – a reasonable assumption based on past findings regarding online and offline jealousy (e.g., Marshall et al., 2013; Hira and Bhogal, 2020) – it appears the association found among those who are relatively confident about online communication is relatively unremarkable. The finding that there is no significant association between attachment anxiety and jealousy among those who have concerns about online communication, therefore, is of particular interest. We can only speculate as to how heightened concern (and possibly apprehension) may reduce the association between attachment anxiety and online jealousy. One possible explanation is that individuals with heightened concern about misunderstandings when communicating online with their partners tend to communicate more in person or to quickly check in with their partners for clarification when confronted by ambiguous online content. Alternatively, or additionally, dating partners of individuals who have heightened concern about misunderstandings may deliberately limit the content of personal messaging and social media posts, thereby reducing opportunities for jealous reactions by their partners.

### Limitations

There are a number of limitations that should be considered when interpreting these findings. First, individuals, rather than couples, were used in this study; studying couples would allow for examination of dyadic processes. Second, the use of hypothetical scenarios to measure jealousy responses precludes generalization to actual jealous-provoking experiences. Third, the attachment measure that was used, while brief and similar in content to more recent measures (Fraley et al., 2000), has less evidence supporting its psychometric properties and does not allow for examining preoccupied/anxious and fearful attachment separately. Fourth, as mentioned, cross-sectional design was used, so causal inferences cannot be inferred. Attachment anxiety and online attitudes were conceptualized as predictor variables in the current study and online jealousy as the outcome variable. While it seems plausible that attachment style, an individual difference variable that is rooted in infancy and stable over time, precedes online attitudes and online jealousy, there is insufficient evidence to determine the causal directions among attachment, attitudes, and jealousy. Fifth, while power analyses indicated that the sample size was sufficient, there is evidence to suggest that interactions may require substantially more power than main effects to detect. It is possible that a larger sample size would detect additional significant interactions (e.g., attachment anxiety and apprehension as predictors of cognitive/behavioral jealousy), or significant differences in correlations between attachment anxiety and type of jealousy, or correlations between men and women. Finally, our sample consisted of college students in dating relationships, thus we must be cautious about generalizing to other types of relationships (e.g., working adults, married couples, etc.).

### Implications and Suggestions for Future Research

As researchers continue to examine relationship dynamics in the digital arena, the current study suggests that it will be important to consider individuals’ attitudes about online communication. In addition to replicating the current findings, further research is needed to clarify how attitudes moderate the impact of attachment anxiety on jealousy; optimal approaches might involve collecting data on the frequency and content of online communication and social media posts for individuals high (and low) in attachment anxiety and their dating partners. Collecting dyadic data will also be important to investigate bidirectional influences among these variables. The exploration of additional factors that may enhance or mitigate online jealousy will likely be useful as well; indeed, there is already evidence that self-esteem (Utz and Beukeboom, 2011), may be influential in predicting online jealousy and other digital relationship processes. Continued development of theory (e.g., attachment theory, the investment model, and self-expansion theory; Rus and Tiemensma, 2017) and the use of a variety of methodological approaches such as observational (e.g., daily diary studies), longitudinal, and experimental designs, are necessary to provide a thorough understanding of relationship processes in the digital arena.

## Data Availability Statement

The raw data supporting the conclusions of this article will be made available by the authors, without undue reservation.

## Ethics Statement

The studies involving human participants were reviewed and approved by Santa Clara University Institutional Review Board. The patients/participants provided their written informed consent to participate in this study.

## Author Contributions

The author confirms being the sole contributor of this work and has approved it for publication.

## Conflict of Interest

The author declares that the research was conducted in the absence of any commercial or financial relationships that could be construed as a potential conflict of interest.

## Footnotes

1 Power analysis (using G^∗^Power 3.1; Faul et al., 2009) indicated that this sample size provided sufficient power to detect an effect size ≥ 0.15, given a *p* value of 0.05 using linear regression with three predictors.
